# Atypical granular cell tumor of the urinary bladder: A case report and literature review

**DOI:** 10.1016/j.eucr.2021.101669

**Published:** 2021-03-29

**Authors:** Antonio Tufano, Vittorio Canale, Giovanni Di Lascio, Giulia Coppola, Rocco Simone Flammia, Cristiano Cristini

**Affiliations:** a Department of Urology, Sapienza University, Rome, Italy; b Department of Radiological, Oncological and Pathological Sciences, Sapienza University, Rome, Italy

**Keywords:** Granular cell tumor, Bladder neoplasm, Bladder tumor

## Abstract

Granular cell tumors (GCTs) are rare neoplasms of neural origin and usually tend to have a benign behaviour. We report a case of a 54 years old woman with severe gross hematuria caused by an atypical granular cell tumor which was successfully managed with a transurethral resection of the bladder (TURB). No local recurrence was observed after a three-year follow-up. The appropriate histological characterization and subclassification (benign, atypical and malignant neoplasm) is mandatory for an optimal patient management, in order to offer an appropriate treatment and a correct follow-up.

## Introduction

Granular cell tumor (GCT) of the bladder is an uncommon soft tissue tumor with a controversial history. This neoplasm typically involves the mucosa of the oral cavity and the tongue, but it can also be found in many different other anatomic sites such as the dermis and subcutaneous tissue. The etiopathogenesis of the GCT is still controversial, but nowadays it diffusely accepted the neural origin from the Schwann cells. Usually, they develop during the fourth or fifth decade and have a slightly higher rate incidence in women. The histological classification, based on morphological and immunohistochemical criteria, subdivides the GCT into three different groups: benign, atypical and malignant. In the majority of cases, it appears in a benign form and only a few display atypical or malignant behavior. We herein report a case that occurred in a 54-year-old woman.

## Case report

**Fig. 1:**
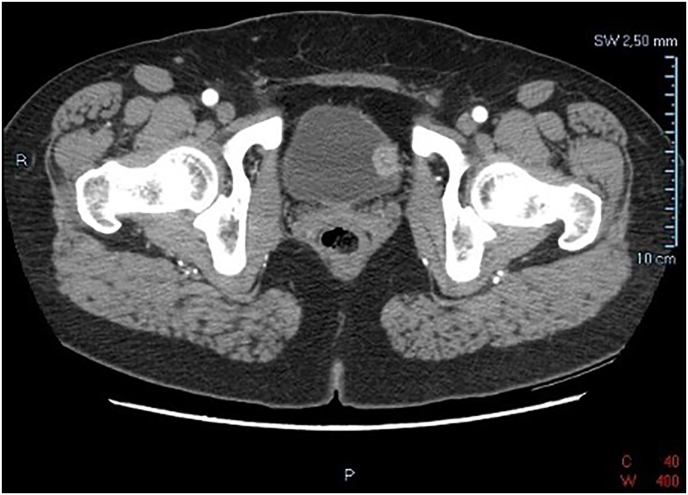
CECT scan showing a 22 mm mass on the left wall of the urinary bladder.

A 54-year-old caucasian woman was referred to the emergency department for progressive lower urinary tract symptoms and hematuria for two months. A hysterectomy due to a large fibroadenoma at the age of forty was also referred. Urinary cytology was negative. Contrast enhanced computed tomography (CECT) revealed a 22 mm mass on the left wall of the bladder (Fig. 1). Cystoscopy confirmed the presence of an exophytic mass on the superior left wall of the bladder. Based on these findings, transurethral resection of bladder tumor (TURB) was performed without early single chemotherapeutical instillation in immediate post-surgery. During the procedure, no active bleeding was observed. The continuous bladder irrigation in the following days showed clear urine and hospitalization lasted for two days.

**Fig. 2:**
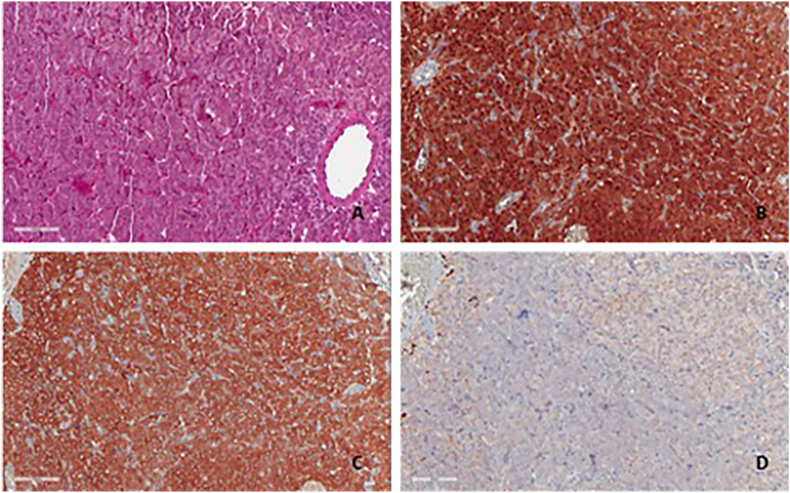
a) The microscopic evaluation shows neoplastic cells with small nuclei and abundant granular eosinophilic cytoplasm (haematoxylin & eosin-stained, x20 magnification). b) Tumor cells show strong and diffuse immunopositivity for S100 (x20 magnification). c) Tumor cells show strong immunopositivity for NSE (x20 magnification). d) Tumor cells are negative for cytokeratin AE1/AE3 (x20 magnification).

Histological evaluation showed multiple fragments of the bladder with normal urothelial cells, with evidence, in the subepithelial connective tissue and on the muscolaris propria of a well circumscribed neoplastic proliferation arranged in nests separated by thin collagenous bands. The neoplastic cells were mostly polygonal and occasionally spindle. Most of the cells presented monomorphic, small round nuclei and abundant cytoplasm containing fine eosinophilic granules (Fig. 2a). The absence of prominent nucleoli was observed. Single scattered cells showing a pleomorphic, voluminous nucleus with eosinophilic nucleoli were also identified. There was no evidence of mitoses or necrosis. The immunohistochemical analysis revealed diffuse positive staining for S100 and NSE (Fig. 2b–c); staining for pan-cytokeratin (CKAE1/AE3) (Fig. 2d) and CD68 were negative. The proliferative index of neoplastic cells evaluated with KI67 was <5%.

Based on these histological and immunohistochemical findings and according to the worldwide used Fanburg-Smith et al. classification,^1^ a diagnosis of atypical granular cell tumor was proposed. Cystoscopy and urinary cytologic exams were performed every 3 months during the first year and every six months during the following 2 years and were always negative. A chest x-ray was also performed every year and did not show any malignancy. The patient is currently disease-free with 3 years of follow up.

## Discussion

GCT frequently originates from the oral cavity and skin, however, the occurrence of GCT in soft tissues and genitourinary system is a relatively rare phenomenon with most cases described on the penis, scrotum and vulva. A review of the literature reported only few cases of bladder GCT, mostly having a benign pattern, nevertheless, they are commonly mistaken for malignant tumors because they are solid-looking, ulcerated and with ill-defined margins. Immunohistochemical studies are particularly useful to differentiate such tumors from carcinomas and sarcomas because GCTs stain strongly and uniformly positive for S-100, enolase neuron specific (NSE), calretinin, the alpha subunit of inhibin, HLA-DR, laminin and various myelin proteins, whereas they are commonly negative for epithelial and sarcoma antigens such as cytokeratin (Cam 5.2 and AE1/AE3), desmin and vimentin. Malignant GCTs (MGCT) tend to have higher cellularity, spindle cells with large nucleoli, numerous atypical mitosis and large areas of necrosis. Cellular variability and pleomorphism alone are not reliable diagnostic criteria of malignancy. Previously reported cases of benign GCTs of the urinary bladder were successfully treated by TURB or by partial cystectomy and only few showed local recurrences during the first year after treatment.^2^ However, the same authors reported that none of these patients, after their last TURB, had evidence of recurrence at 2.5 years follow up. Conversely in the malignant form, the local recurrence is common, and the prognosis is poor.

To our knowledge, only two cases of MGCT of the bladder have been described so far^3^^,^^4^; the first was managed with a complete excision of the mass, however the patient during the 17 months of follow up developed distant metastases and died. In the second patient the tumor was treated with radical cystectomy, hysterectomy with bilateral salpingo-oophorectomy, anterior vaginectomy, and lymph node dissection, showing a long-term disease-free survival.

In the review by Fanburg-Smith^1^ et al., which presented a series of 28 patients with MGCT of soft tissue, local recurrence was observed in 32%, metastasis in 50% and 39% died within 3 years of the initial diagnosis. The average size of metastatic tumors was 5 cm and the time intercurred between the diagnosis and the metastasis was 2 years. Older age was the only significant variable associated with the development of metastases.

We describe the first atypical GCT case of the bladder and data on other anatomical sites are extremely limited. The classification of atypical is still under debate, Machado and colleagues,^5^ indeed, proposed to abandon the distinction between benign and atypical GCTs and to designate both categories as “GCT with almost no metastatic potential”, because most cases previously classified as atypical in the literature did not metastasize, and only some recurred locally, similar to incompletely excised benign tumors.

## Conclusion

Granular cell tumors (GCTs) are extremely uncommon lesions. We recommend a careful histological examination for establishing the appropriate diagnosis and treatment. TURB is appropriate for benign cases in contrast to a more radical approach for a malignant tumor. Atypical neoplasm treatment is still not well defined. In our case, a conservative approach seems to be safe although, further studies with long-term follow-up may be indicated to confirm this impression.
